# Obesity is negatively associated with dental caries among children and adolescents in Huizhou: a cross-sectional study

**DOI:** 10.1186/s12903-022-02105-5

**Published:** 2022-03-17

**Authors:** Rou Shi, Chunwen Lin, Shu Li, Linling Deng, Zhan Lin, Liangchang Xiu

**Affiliations:** 1 Department of Endocrinology, Huizhou Municipal Central Hospital, Huizhou, 516001 Guangdong China; 2 Department of Epidemiology and Medical Statistics, Guangdong Medical University, Dongguan, 523808 Guangdong China

**Keywords:** Dental caries, Obesity, Adolescents, Children

## Abstract

**Background:**

Obesity and dental caries among children and adolescents are growing worldwide public health problems. They share some common and modifiable influences. The objective of this study was to evaluate the prevalence of obesity and dental caries among children and adolescents in Huizhou and explore the association between Body Mass Index (BMI) category and dental caries.

**Methods:**

This cross-sectional study enrolled 105,181 students (55,500 males and 49,681 females) from 87 schools in Huizhou. Height and weight were measured, and BMI was calculated. Based on Chinese BMI standards, students were classified into underweight, normal weight, overweight, and obesity groups. Dental caries was diagnosed according to criteria recommended by World Health Organization (WHO). We used the Chi-square test to compare proportions of groups and performed Association Rules Mining to identify patterns and combinations of BMI categories and dental caries. Finally, a multilevel logistic regression model was applied to analyze the association between BMI category and dental caries when confounders were considered.

**Results:**

The prevalence of underweight, overweight, and obesity among children and adolescents was 7.56%, 8.85%, and 2.95%, respectively. The overall prevalence of dental caries was 58.10%, with a lower prevalence among boys than girls. Students from primary schools and remote towns more easily suffer from dental caries. Caries prevalence of students belonged to underweight, normal, overweight, and obesity was 65.6%, 58.8%, 49.6%, and 46.1% individually. With increasing BMI levels, the prevalence of dental caries decreased. Further, this trend still exists in each subgroup of gender, educational stage, school type, and area. Association rules indicate underweight has a positive effect on the occurrence of dental caries, while overweight or obesity has a negative impact on the occurrence of dental caries. The three-level logistic regression model results show that BMI category is inversely associated with dental caries after adjusting confounders.

**Conclusion:**

Obesity is negatively associated with dental caries among children and adolescents in Huizhou. Further research is required to investigate how dietary habits, oral hygiene habits, and parental socioeconomic status mediate the association between BMI and dental caries.

## Background

In recent years, the prevalence of obesity among children and adolescents has continued to increase globally. More than 340 million children and adolescents were overweight or obese in 2016 worldwide [1]. The proportion has risen more than four-fold from 4% in 1975 to 18% in 2016 [2]. Studies have revealed that obese children and adolescents are likely to maintain their status into adulthood and are at higher risk of developing chronic diseases, including hypertension, type 2 diabetes, heart disease, cancer, and so on [3–5]. Many factors, such as high birth weight, post-natal overfeeding, and high-energy diets, are thought to be strongly associated with childhood and adolescence obesity [6–8]. Preventing and managing obesity in children and adolescents will be a great challenge [2, 9]. A broad understanding of the obesity epidemic in children and adolescents will help guide intervention efforts and develop effective population-based programs and policies.

Dental caries, which remains a severe public issue worldwide, is also one of the most common diseases, especially in many developing countries [10, 11]. Kazeminia [12] reviewed 164 articles on the prevalence of dental caries from 1995 to 2019 and found that caries prevalence of primary teeth and permanent teeth in children in the world was 46.2% and 53.8%, respectively. Obesity and dental caries share some common, modifiable factors such as diet and lifestyle. BMI is widely used as a surrogate measure for obesity and is a commonly used indicator for nutritional status as well. Given that both caries prevalence and BMI measure diet-related health outcomes, a relationship between BMI and dental caries seems logical. Numerous studies have investigated the association between BMI and dental caries. However, the results have always been controversial and inconclusive. Both a low and a high BMI were associated with dental caries in Nepalese schoolchildren [13]. But there was no association between obesity and dental caries among Korean adolescents [14]. Even in different provinces of China, the results were inconsistent. Yao [15] thought BMI categories were positively associated with dental caries in the Wannan area, Anhui Province. Li [16] reported overweight and obesity status were negatively related to dental caries among school children and adolescents in Jiangsu Province. The systematic review and meta-analysis results were also conflicting [17, 18]. We consider there are two main reasons for the difference in results. First, the development of obesity has many factors and complexity [19, 20]. It is not only related to age, sex, exercise, diet, and individual mood but also varies by race and ethnicity [2, 21]. Second, the sample size of most of the studies is small, lacking representativeness, and there may be a selection bias. While many countries collect data on children under five years of age through large-scale surveys, there is a lack of research on older children and adolescents, and the sample sizes tend to be smaller [22].

The purpose of this study is to estimate the prevalence of obesity and dental caries among children and adolescents in Huizhou and to explore the association between BMI category and dental caries after adjusting gender, age, educational stage, school type, and so on.

## Methods

### Research methodology

A cross-sectional study was conducted during the 2019–2020 academic year, part of the surveys on students' constitution and health carried out by the government in Huizhou, Guangdong Province, China.

### Sampling

The subjects were sampled by the multistage stratified cluster sampling method. In the first stage, all towns in Huizhou were divided into three strata according to economic levels. Then, 13 towns were randomly selected. Five were from the urban area, six suburban area, and two were from remote towns. In the second stage, schools of each selected town were partitioned into public schools and private schools. Afterward, eighty-seven schools, including primary school (grade 1–6, 7–12 years old), junior high school (grade 7–9, 13–15 years old), and senior high school (grade 10–12, 16–18 years old) were randomly selected, ensuring proportionate representation of different economic levels. Finally, students in the selected schools were recruited as study subjects. Written informed consent was obtained from all subjects' parents. The research was approved by the Clinical Research Ethics Committee of Huizhou People's Hospital.

### Inclusion and exclusion criteria

Inclusion criteria were: (1) students over seven years of age in primary school, junior high school, and senior high school; (2) living in the sampling area for more than six months. The exclusion criteria were as follows: (1) suffering from obvious diseases (like hepatitis, tuberculosis, etc.) or physical/mental deformities (such as physical disability or malformation); (2) absence from examination or missing anthropometric data. Finally, 105,181 students (93.2%) of a total of 112,807 were included in this cross-sectional study.

### Measurements

Four teams performed the anthropometric measurement and oral examinations. Each group consisted of two nurses, a physician, and a dentist. To ensure study reliability, a training and calibration process was carried out. They were trained with Technical Standard for Physical Examination for Students (National Standards of the People’s Republic of China, GB/T-26343-2010) [23]. The height meter and balance weight scale were all calibrated before measuring. The measurement error should not exceed ± 0.5 cm for height and ± 0.1 kg for weight. Students were asked to dress in light clothing and barefoot when height and weight were measured. Blood pressure was measured three times for each student at 1-min intervals, and the mean was used in the analysis.

Dentists examined the teeth of each student by using a flat mouth mirror and a CPI probe. Caries was diagnosed according to WHO criteria, and caries experience was measured as the DMFT (number of decayed, missing, and filled permanent teeth). In contrast, the dmft was used for primary teeth. Dentists were calibrated for diagnosing decayed teeth, and the degree of agreement was assessed. The kappa coefficients were more than 0.80 for all examiners. Demographic information, including age, sex, residence, grade, and school type, was also collected.

### Definitions

BMI was calculated by dividing their weight (in kilograms) by their height (in meters) squared. Participants were categorized as underweight, normal weight, overweight, and obese according to the sex- and grade-stratified cutoff values of BMI in National Student Physical Health Standards revised by the Ministry of Education in 2014 [24]. Underweight is defined as under the 5th percentile curve, normal weight as between 5 and 85th percentile, overweight as higher than 85th and lower than the 95th percentile, and obesity as higher or equal to the 95th percentile. The presence of caries was diagnosed if dmft or DMFT > 0.

### Statistical analysis

Conventional descriptive statistics described data. Continuous variables were summarized based on mean ± SD, while categorical variables were summarized based on frequency (percentage). The two or more groups were compared for proportions using the Chi-square test. Association Rules Mining was performed to identify patterns and combinations of BMI categories and dental caries. The ARM is a fundamental data mining technique that exhaustively looks for hidden patterns, making them ideal for discovering predictive rules from medical databases. An association rule (AR) is a pair (X, Y) of sets of attributes, denoted by X → Y, where X is the antecedent and Y is the consequent of the rule X → Y. Basically, the rule states that if X happens, then Y does happen. In general, a set of items, such as X or Y, which are disjoint, is called an item set. Applied to a medical condition, association rules can identify subgroups at exceptionally high risk of a given disease. We employed the three commonly used measurement ratios: support (how frequently the disease combinations appear in the data set), confidence (the conditional probability that a participant who has the antecedent disease will also have the consequent illness), and lift (the ratio of the observed support to that expected if the two events were independent). The lift measures the importance of a rule within ARM, so it was considered the primary measure of significance in our study [25].

In this study, data had a multilevel or hierarchical structure. Students learned in different grades; each school contained multiple grades; we sampled schools of different types in different regions. Units at one level were recognized as being nested within units at the next higher level. Students in the same grade were more similar than those in different grades within a school, and students within a school would be more alike, on average, than students from different schools. A three-level logistic regression model (the level 1 units were individual students, the level 2 units were grades, and the level 3 units were schools) was fitted to explore the association between BMI category and dental caries when other variables were also considered. The model was estimated using iterative generalized least squares (IGLS), and the Wald test was used to test the significance of coefficients.

All analyses were performed with SPSS v25.0 (IBM SPSS Statistics for Windows, IBM Corp, Armonk, NY) and MLwin Version 2.36 (Centre for Multilevel Modelling, University of Bristol). *P*-value < 0.05 was considered as statistical significance.

## Results

### Characteristics of subjects

A total of 105,181 students, including 55,500 males (52.77%) and 49,681 females (47.23%) were enrolled in the present data. The gender composition significantly differed between educational stages $(\chi^{2} = 147.86, P < 0.001)$. More boys than girls were in primary schools and junior high schools, while more girls than boys in senior high schools. 67,206 students (63.90%) were from public schools, 37,975 (36.10%) were from private schools. The proportions of students from public schools and urban areas increased rapidly with the educational stage up. The age (mean ± SD) was $12.64 \pm 3.30$ years. More details are shown in Table 1.

**Table 1:** Characteristics of participants

Characteristics	Primary school		Junior high school		Senior high school		Total	
	*n*	%	*n*	%	*n*	%	*n*	%
Gender								
Male	26,684	53.90	18,334	53.42	10,482	49.08	55,500	52.77
Female	22,823	46.10	15,984	46.58	10,874	50.92	49,681	47.23
School type								
Public school	25,127	50.75	22,114	64.44	19,965	93.49	67,206	63.90
Private school	24,380	49.25	12,204	35.56	1391	6.51	37,975	36.10
Area								
Urban	29,160	58.90	27,959	81.47	21,062	98.62	78,181	74.33
Suburb	15,012	30.32	6019	17.54	294	1.38	21,325	20.27
Remote town	5335	10.78	340	0.99	0	0.00	5675	5.40
Age*	9.71 ± 1.75		14.01 ± 0.97		17.22 ± 0.99		12.64 ± 3.30	
Height (cm)*	133.84 ± 11.67		160.20 ± 7.96		166.16 ± 8.16		149.00 ± 17.51	
Weight (kg)*	29.80 ± 8.48		49.81 ± 9.26		56.20 ± 9.39		41.56 ± 14.46	
SBP (mmHg)*	101.76 ± 9.16		110.72 ± 11.28		114.14 ± 11.30		107.26 ± 12.42	
DBP (mmHg)*	66.12 ± 7.00		70.80 ± 8.20		72.24 ± 8.15		68.91 ± 8.13	

*$\overline{x} \pm s$; SBP, systolic blood pressure; DBP, diastolic blood pressure

### Outcome of BMI classification

**Fig. 1:**
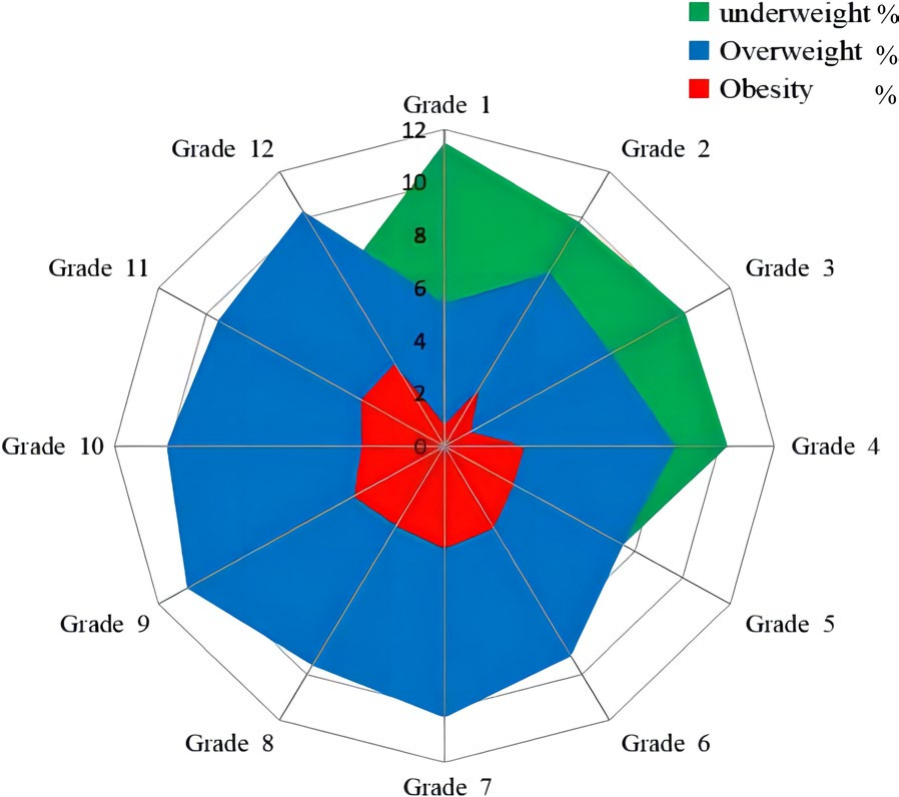
The prevalence of underweight, overweight and obesity among children and adolescents in different grades

**Fig. 2:**
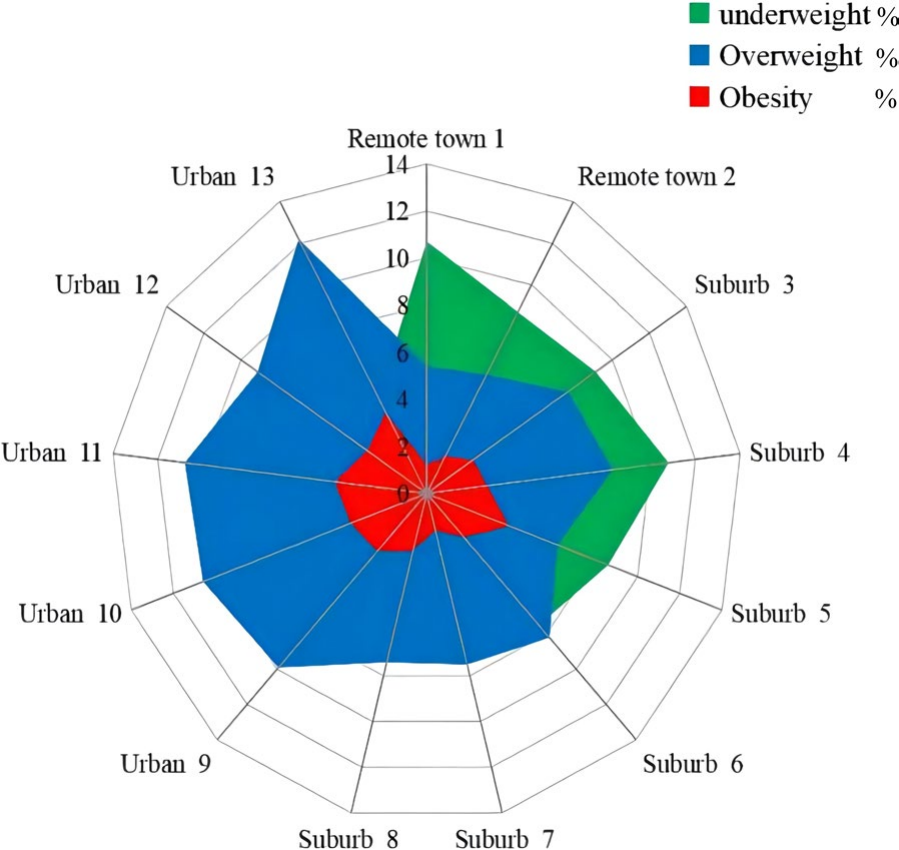
The prevalence of underweight, overweight and obesity among children and adolescents in selected towns

BMI classification of the study subjects and subgroups was summarized in Table 2. The prevalence of underweight, overweight, and obesity for all was 7.56%, 8.85%, and 2.95%, respectively. More boys were skinny (7.84% vs. 7.24%) and overweight (9.45% vs. 8.17%) than girls. In comparison, obesity was slightly higher in girls than boys (3.15% vs. 2.76%). About 9.46% of primary school pupils were categorized as underweight, greater than junior high school students and senior high school students. Conversely, junior high school students and senior high school students were more easily overweight and obese. The frequencies of underweight, overweight, and obesity in different grades were displayed in Fig. 1, which showed that underweight, overweight, and obesity mainly concentrate in grade 1–4, grade 7–12, and grade 7–9, respectively. Students from remote towns had a greater prevalence of underweight, and urban area students were more likely to be overweight and obese. Figure 2 depicts the frequencies of underweight, overweight, and obesity in different towns.

**Table 2:** BMI classification of children and adolescents

Variables	Underweight (*n* = 7948)	Normal (*n* = 84,831)	Overweight (*n* = 9304)	Obesity (*n* = 3098)	Total (*n* = 105,181)
Gender					
Male	4350 (7.84)	44,373 (79.95)	5247 (9.45)	1532 (2.76)	55,500
Female	3598 (7.24)	40,460 (81.44)	4057 (8.17)	1566 (3.15)	49,681
Educational stage					
Primary school	4682 (9.46)	40,056 (80.91)	3670 (7.41)	1099 (2.22)	49,507
Junior high school	1943 (5.66)	27,586 (80.38)	3513 (10.24)	1276 (3.72)	34,318
Senior high school	1323 (6.19)	17,189 (80.49)	2121 (9.93)	723 (3.39)	21,356
School type					
Public	4883 (7.27)	53,884 (80.18)	6325 (9.41)	2114 (3.15)	67,206
Private	3065 (8.07)	30,947 (81.49)	2979 (7.85)	984 (2.59)	37,975
Area					
Urban	3082 (6.04)	41,029 (80.37)	5157 (10.10)	1781 (3.49)	51,049
Suburb	4290 (8.85)	39,090 (80.67)	3836 (7.92)	1241 (2.56)	48,457
Remote town	576 (10.15)	4712 (83.03)	311 (5.48)	76 (1.33)	5675

### Prevalence of dental caries

Caries prevalence of children and adolescents according to gender, educational stage, school type, area, and BMI categories were presented in Table 3. The overall prevalence of dental caries was 58.10%, with a lower prevalence among boys than girls (54.92% vs. 61.65%, $\chi^{2} = 488.64, P < 0.001$). Significant differences in caries status were also found across educational stage (71.85% vs. 47.07% vs. 43.95, $\chi^{2} = 7318.17, P < 0.001$), school type (55.68% vs. 62.38, $\chi^{2} = 447.01, P < 0.001$) and area (49.49% vs. 64.27% vs. 82.93%, $\chi^{2} = 3749.31, P < 0.001$). Students from primary schools, private schools, and remote towns more easily suffer from dental caries.

**Table 3:** Caries prevalence of the study subjects and subgroups

Variables	Underweight		Normal		Overweight		Obesity		Total	
	Caries (*n* = 5211)	Caries free (*n* = 2737)	Caries (*n* = 49,858)	Caries free (*n* = 34,973)	Caries (*n* = 4613)	Caries free (*n* = 4691)	Caries (*n* = 1427)	Caries free (*n* = 1671)	Caries (*n* = 61,109)	Caries free (*n* = 44,072)
Gender										
Male	2709 (62.28)	1641 (37.72)	24,812 (55.92)	19,559 (44.08)	2319 (44.20)	2928 (55.80)	639 (41.71)	893 (58.29)	30,479 (54.92)	25,021 (45.08)
Female	2502 (69.54)	1096 (30.46)	25,046 (61.90)	15,414 (38.10)	2294 (56.54)	1763 (43.46)	788 (50.32)	778 (49.68)	30,630 (61.65)	19,051 (38.35)
Educational stage										
Primary school	3566 (76.16)	1116 (23.84)	29,074 (72.58)	10,982 (27.42)	2298 (62.62)	1372 (37.38)	633 (57.60)	466 (42.40)	35,571 (71.85)	13,936 (28.15)
Junior high school	991 (51.00)	952 (49.00)	13,177 (47.77)	14,409 (52.23)	1472 (41.90)	2041 (58.10)	513 (40.20)	763 (59.80)	16,153 (47.07)	18,165 (52.93)
Senior high school	654 (49.43)	669 (50.57)	7607 (44.26)	9582 (55.74)	843 (39.75)	1278 (60.25)	281 (38.87)	442 (61.13)	9385 (43.95)	11,971 (56.05)
School type										
Public school	3110 (63.69)	1773 (36.31)	30,325 (56.28)	23,559 (43.72)	3047 (48.17)	3278 (51.83)	939 (44.42)	1175 (55.58)	37,421 (55.68)	29,785 (44.32)
Private school	2101 (68.55)	964 (31.45)	19,533 (63.12)	11,414 (36.88)	1566 (52.57)	1413 (47.43)	488 (49.59)	496 (50.41)	23,688 (62.38)	14,287 (37.62)
Area										
Urban	1696 (55.03)	1386 (44.97)	20,555 (50.10)	20,474 (49.90)	2274 (44.10)	2883 (55.90)	737 (41.38)	1044 (58.62)	25,262 (49.49)	25,787 (50.51)
Suburb	3018 (70.35)	1272 (29.65)	25,371 (64.90)	13,719 (35.10)	2114 (55.11)	1722 (44.89)	638 (51.41)	603 (48.59)	31,141 (64.27)	17,316 (35.73)
Remote town	497 (86.28)	79 (13.72)	3932 (83.45)	780 (16.55)	225 (72.35)	86 (27.65)	52 (68.42)	24 (31.58)	4706 (82.93)	969 (17.07)

**Fig. 3:**
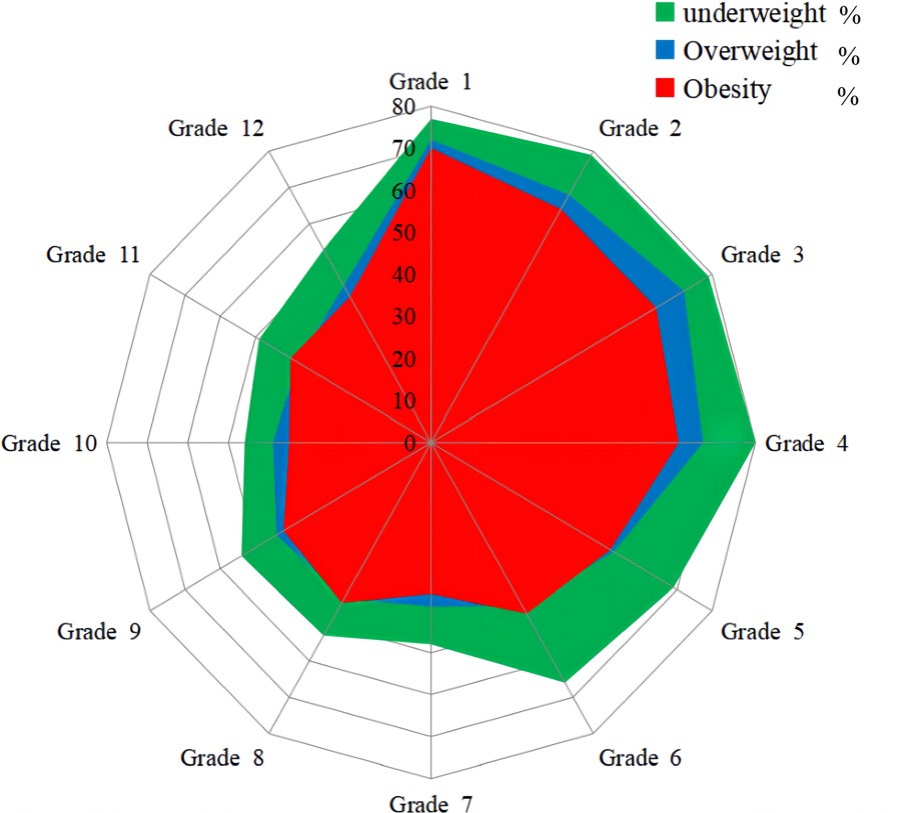
The prevalence of dental caries among children and adolescents with different BMI categories in each grade

**Fig. 4:**
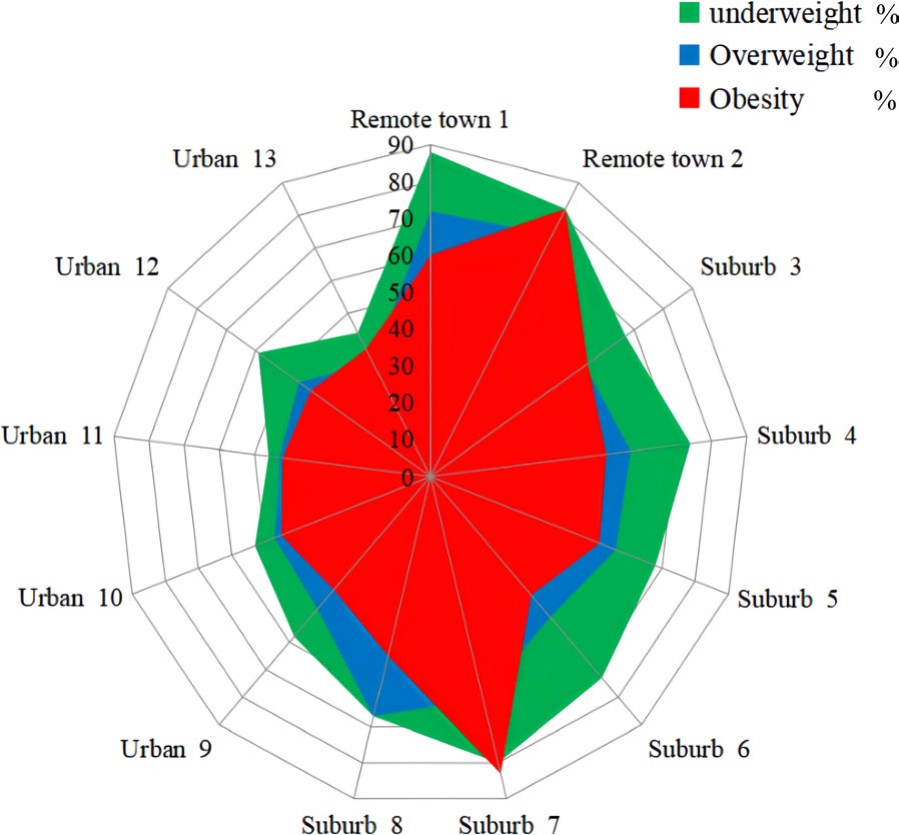
The prevalence of dental caries among children and adolescents with different BMI categories in selected towns

Caries prevalence of students belonged to underweight, normal, overweight, and obesity was 65.6%, 58.8%, 49.6%, and 46.1%, respectively. With increasing BMI levels, the prevalence of dental caries decreased (Cochran Armitage trend test *Z* = 25.33, *P* < 0.001). Further, this trend still exists in each subgroup of gender, educational stage, school type, and area. Caries prevalence of students with the same BMI level declines as the grade increases. In almost all grades, the maximum value of the caries prevalence belonged to underweight, followed by overweight, and the minimum value was obesity (Fig. 3). Figure 4 depicts the caries prevalence of each town. In the case of adjusting the BMI effect, students from urban schools still have the lowest caries prevalence, and in almost all towns, with the BMI level up, caries prevalence also comes down.

### Association rules

The ARM Results were listed in Table 4, showing patterns of BMI categories and dental caries. The probability for underweight and dental caries in all subjects was 4.95%, normal and dental caries was 47.40%. Further, 4.39% of students with overweight also reported dental caries, whereas1.37% of those with obesity also reported dental caries. Confidence of association rules revealed the probabilities of dental caries in underweight, normal, overweight, or obesity, which were the same as we mentioned above (3.3 Dental caries among children and adolescents). The lift values were 1.13, 1.01, 0.85, and 0.79 for underweight and dental caries, normal weight and dental caries, overweight and dental caries, obesity and dental caries, respectively.

**Table 4:** Association rules of BMI category and dental caries

Rule		Support (%)	Confidence (%)	Lift
Condition	Result			
Underweight	Dental caries	4.95	65.56	1.13
Normal	Dental caries	47.40	58.77	1.01
Overweight	Dental caries	4.39	49.58	0.85
Obesity	Dental caries	1.37	46.06	0.79

### Associations between BMI category and dental caries under confounders adjusted

**Fig. 5:**
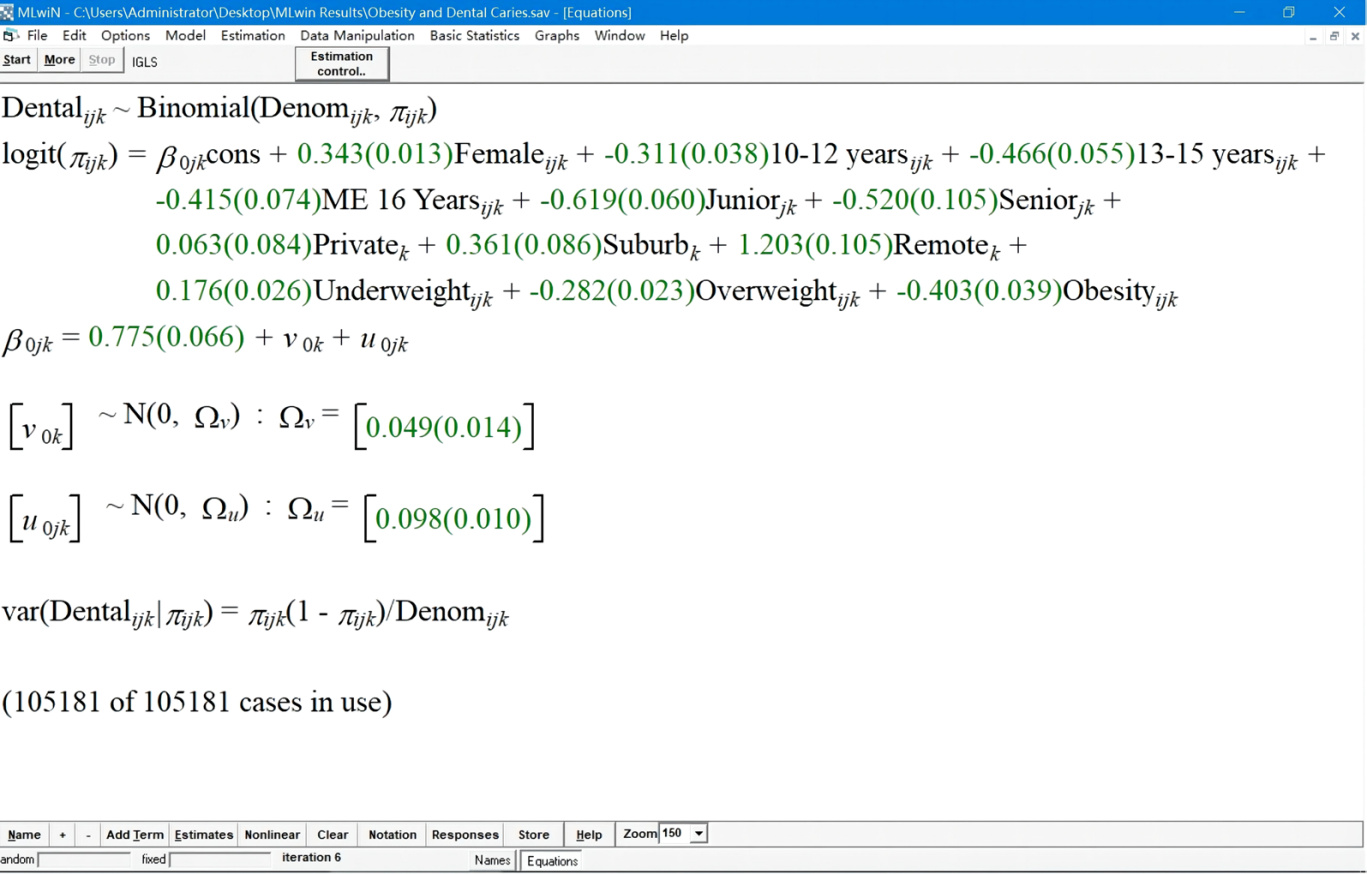
The fitted three-level logistic regression model

The fitted three-level logistic regression model was shown in Fig. 5, and the estimates and *ORs* were demonstrated in Table 5. Gender, age, educational stage, area, and BMI category were significantly associated with dental caries. Girls were more likely to have caries than boys (*OR* = 1.41, 95% CI 1.37–1.45). Compared with younger pupils, older students were less likely to suffer from dental caries, and similar differences were found between educational stages. Students from suburbs and remote towns were 1.43 (*OR* = 1.43, 95% CI 1.21–1.70) and 3.33(*OR* = 3.33, 95% CI 2.71–4.09) times more likely to have dental caries than those from urban areas. Using normal weight as a reference, the odds for the prevalence of dental caries increased 19% (*OR* = 1.19, 95% CI 1.13–1.26) in underweight students and decreased by 25% (*OR* = 0.75, 95% CI 0.72–0.79) and 33% (*OR* = 0.67, 95% CI: 0.62–0.72) in overweight and obesity students, respectively. BMI category has an inverse association with dental caries after adjusting confounders.

**Table 5:** Results of multivariable logistical regression model

Variables	*OR*	95% CI		*P*
Gender				
Male	1.00			
Female	1.41	1.37	1.45	< 0.0001
Age				
≤ 9	1.00			
10–12	0.73	0.68	0.79	< 0.0001
13–15	0.63	0.56	0.70	< 0.0001
≥ 16	0.66	0.57	0.76	< 0.0001
Educational stage				
Primary school	1.00			
Junior high school	0.54	0.48	0.61	< 0.0001
Senior high school	0.59	0.48	0.73	< 0.0001
School type				
Public school	1.00			
Private school	1.07	0.90	1.26	0.4523
Area				
Urban	1.00			
Suburb	1.43	1.21	1.70	< 0.0001
Remote town	3.33	2.71	4.09	< 0.0001
BMI				
Normal	1.00			
Underweight	1.19	1.13	1.26	< 0.0001
Overweight	0.75	0.72	0.79	< 0.0001
Obesity	0.67	0.62	0.72	< 0.0001

## Discussion

This study evaluated the prevalence of obesity and dental caries among children and adolescents in Huizhou and explored the association between BMI category and dental caries. The principal findings are as follows: (1) The prevalence of overweight and obesity was 8.85% and 2.95%, respectively; (2) The overall prevalence of dental caries was 58.10%, with a lower prevalence among boys than girls. Students from primary schools, private schools, and remote towns more easily suffer from dental caries; (3) With increasing BMI levels, the prevalence of dental caries decreased. This trend still exists in each subgroup of gender, educational stage, school type, and area. (4) There was an inverse association between BMI category and dental caries after adjusting for confounders. In other words, obesity is negatively associated with dental caries.

### The prevalence of overweight and obesity

Data from the 2017 Physical Activity and Fitness in China show that the prevalence of overweight and obesity among Chinese school-aged children and adolescents is 15.1% and 10.7%, respectively [26]. So, Huizhou’s overweight and obesity prevalence rate is lower than the national level. There is a geographic disparity in childhood obesity prevalence in China, higher in the northern region while lower in the southern part [27]. This geographic variation may be attributed to the differences in dietary habits, climate, genetics, etc. Huizhou is in the southeast of Guangdong province, a coastal city with a mean maximum air temperature of 28 °C. Cantonese are used to drinking slow-cooked soup and paying more attention to a healthy diet, and even in the winter, children can have a long time doing outdoor activities.

### The prevalence of dental caries

Caries prevalence of children and adolescents in Huizhou (58.10%) was higher than that of a population-based study in Shenzhen (41.15%) [28] and an extensive survey in Guangzhou (30.7%) [29]. The reasons may as follow: firstly, Huizhou has a lower socioeconomic level than Shenzhen and Guangzhou. Secondly, on average, the parental education level of students in Huizhou is also lower than that of students in Shenzhen and Guangzhou. Lastly, the researchers only selected the children in urban areas in Guangzhou. Low levels of parental income and education were associated with dental caries [30]. Caries prevalence increased with the decreasing socioeconomic status (SES) [14, 31], which may also explain the difference of caries prevalence in areas. Female students have a higher caries prevalence than male students. This finding is consistent with the results in other provinces of China [32, 33]. One possible explanation could be girls' preference for cariogenic foods, in addition to earlier tooth eruption in girls compared to boys, thus exposing their teeth to a cariogenic oral environment for a longer time than boys [33].

### The association between BMI category and dental caries

Obesity and dental caries are a comorbidity in many populations because of some common risk factors, including consumption of highly caloric and cariogenic foods. The current data show that the prevalence of dental caries is significantly lower in overweight and obese students but higher in underweight students, taking normal ones as reference. There has been conflicting evidence in the literature about the nature and direction of this association. Alshihri et al. [34] reviewed papers (published from 2015 to 2018) on the association between obesity and dental caries in children and adolescents, and found that of the twenty-six included studies, nine studies reported no relationship between obesity and dental caries, five studies declared a positive association, while eleven studies observed an inverse association like our results.

The possible reasons for the differences may be as follows: (1) The target populations are different. The studies were conducted in different countries or regions [13, 15, 29, 35]. There may be population heterogeneity. For example, Hayden et al. [14] moderated the study country of origin (newly industrialized versus industrialized). They found a significant relationship between obesity and dental caries in children from industrialized but not freshly industrialized countries. (2) Different classification criteria produce different groups. Some studies adopted the recommended age- and gender-specific WHO growth references, expressed as z-scores and categorized into four subgroups: underweight, normal, overweight, and obese [36, 37]. Several studies employed the Centers for Disease Control and Prevention (CDC) and categorized subjects into four subgroups as well [38, 39]. Other studies used the international BMI recommended by the International Obesity Task Force (IOTF), which separates samples into two categories, “not overweight” and “overweight” [40]. (3) Differences in sample size and variation in the confounders used for adjustment (such as gender, age, socioeconomic and residence area) may also cause different results. Some studies recruited hundreds of children [41], while the most extensive study enrolled millions [28].

Our study supported a negative association between BMI category and dental caries. More overweight and obese children and adolescents come from economically wealthy families and have parents with higher education in China. The role of SES is opposite to that in developed countries. Parents in the urban area spend more money on food, and most of them still believe that higher food intake makes their children grow faster. This results in overweight urban children and adolescents [42]. These parents also pay more attention to their child’s health status, including oral health [29]. Among children of high SES families, overweight children had approximately 71% fewer caries than those who were normal weight [43].

Contrarily, underweight usually means under-nutrition. Enamel hypoplasia, salivary glandular hypofunction, and saliva compositional changes may be how malnutrition is associated with caries [44]. On the other hand, untreated caries might cause severe pain and discomfort in children, thus reducing food intake. In addition, other symptoms induced by caries, including infection, irritability, and disturbed sleeping habits, can affect children's quality of life and, thereby, underweight [45]. In the future, a cohort study, collecting more information such as dietary habits, oral hygiene habits, and SES of students will be helpful to clarify the causal relationship between underweight and dental caries.

### Strength and limitation

The strengths of this study include a large sample size, wide age range, and the inclusion of both sexes, and the use of National Standards to measure. Also, we used stratified analysis, Association Rules Mining and multilevel Logistic regression model to confirm the association between BMI category and dental caries. However, some potential limitations also warrant discussion. First, no causal association can be made as with any cross-sectional study. Second, other factors, including dietary habits, oral hygiene habits, and parental SES, have also been observed to exert significant influences on the occurrence of dental caries and obesity. We have not evaluated those confounders in our data yet. Therefore, residual confounding could bias the observed associations. Finally, since the sample referred to Huizhou children and adolescents, more work is needed to evaluate whether these results extrapolate to other racial or ethnic groups.

## Conclusion

In conclusion, we found a lower prevalence of overweight and obesity but a higher prevalence of dental caries among children and adolescents in Huizhou, China. With the BMI levels increasing, the occurrence rate of dental caries decreases. The findings could help us further understand the relationship between BMI category and dental caries and could be the basis of policy for preventing obesity and dental caries in children and adolescents. In the future, cohort studies are required to confirm the present study’s results and clarify the mechanisms of the association between BMI and dental caries.

## Data Availability

All data generated or analyzed during this study are included in this article and its supplementary material files. Further inquiries can be directed to the corresponding author. It is available from the corresponding author on reasonable request.

## Abbreviations

BMI: Body Mass Index
WC: Waist circumference
DMFT: Decayed, missing or filled teeth
WHO: World Health Organization
SD: Standard Deviation
SBP: Systolic blood pressure
DBP: Diastolic blood pressure
IOTF: International Obesity Task Force
SES: Socioeconomic Status
